# Effects of Fentanyl-Adulterated Methamphetamine on Circulating Ghrelin in Rats

**DOI:** 10.3390/ijms262411806

**Published:** 2025-12-06

**Authors:** Huimei Wei, Elise C. Maul, Shawn Park, Kaniz Fatema, Daniel J. Peter, Chang-Guo Zhan, Fang Zheng

**Affiliations:** 1 Molecular Modeling and Biopharmaceutical Center, College of Pharmacy, University of Kentucky, 789 South Limestone Street, Lexington, KY 40536, USA; 2 Department of Pharmaceutical Sciences, College of Pharmacy, University of Kentucky, 789 South Limestone Street, Lexington, KY 40536, USA

**Keywords:** opioid, fentanyl, methamphetamine, ghrelin, polydrug use

## Abstract

The appetite hormone ghrelin influences biological processes that are responsible for substance use disorder, which is related to alcohol and most abused drugs including cocaine, methamphetamine, nicotine, etc. In general, upregulation of the ghrelin system enhances drug cravings and substance use. Studies reported in the literature consistently demonstrated that the ghrelin system is associated with stimulants. However, research on opioids in combination with methamphetamine has not been reported. In this study, we examined the relationship of circulating ghrelin with the polydrug use of fentanyl and methamphetamine in male Sprague-Dawley rats, demonstrating for the first time that concurrent use of fentanyl and methamphetamine significantly increased plasma acyl-ghrelin (the active form of ghrelin) and total ghrelin concentrations. Additionally, the data also demonstrated for the first time that the use of fentanyl alone also significantly increased the plasma ghrelin concentrations. These findings imply that the ghrelin system could be a potential pharmacological target for the treatment of substance use disorders caused by polydrug use involving fentanyl and methamphetamine as well as the fentanyl use alone.

## 1. Introduction

Fentanyl is a synthetic opioid used as a prescription drug for the management of severe pain with a ~100 times stronger potency than morphine [1]. Like other opioids, fentanyl may cause negative health effects such as drowsiness, pinpoint pupils, respiratory depression, etc., of which two milligrams can be lethal to humans [2]. In recent years, illicit fentanyl is being distributed in the illegal drug market, significantly contributing to the opioid epidemic in the United States [2,3].

Fentanyl is often mixed with other illicit drugs to increase the potency [4,5]. This is because fentanyl is cheaper and addictive, which easily makes people addicted and keeps them coming back for more at a low cost. There are growing concerns in society on widespread contamination of stimulants with fentanyl [6,7,8]. In 2019, 8.4% of methamphetamine (METH)-positive samples of urine were tested positive for fentanyl by a urine testing company [9]. A more recent study indicated that prevalence of fentanyl in powder methamphetamine was 12.5% [10]. It was also reported that co-use of METH and opioids hinders opioid use disorder (OUD) treatment [4]. There was a surging rise in METH use in chronic opioid users; past month use of METH significantly increased among treatment-seeking opioid users (+82.6%, *p* < 0.001), from 18.8% in 2011 to 34.2% in 2017 [11]. The METH-opioid combination produces a greater rewarding effect than the opioid or METH alone [12]. Particularly, the METH-fentanyl combination is recognized as one of the most widely used poly-drugs driving the current wave of overdose crisis in the United States [13] and, hence, the goal of our present study is mainly focused on the METH-fentanyl polydrug.

METH is known as a stimulant that activates the central nervous system (CNS), while fentanyl is one of the opioids recognized as CNS depressants, and certain pharmacological effects of METH and fentanyl may conceal one another [13]. It is interesting to understand the reasons leading to drug abuse of the combination. So far, a considerable number of research studies have suggested that ghrelin is involved in the neurobiological processes behind drug use and addiction, which is related to most abused substances including alcohol, cocaine, METH, nicotine, etc. [14,15,16]. Ghrelin is an appetitive hormone that signals the brain to eat when the stomach is empty [17]. Ghrelin receptors are found in many brain areas, including the ventral tegmental area (VTA), which is involved in reward [14]. The behavioral and biological outcomes of some of the most abused drugs were manipulated by ghrelin receptor antagonism, which in turn was explored for drug abuse treatment [15,16,18,19,20,21]. In general, ghrelin upregulation enhances craving for drugs and substances use [15]. For example, administration of METH or cocaine alone significantly increased total ghrelin blood levels in rats [22,23,24,25,26]. Research outcomes reported in the literature consistently demonstrated that the ghrelin system is associated with stimulants, while research on opioids in combination with METH has not been reported. In this study, we explored the relationship between circulating ghrelin and the combined use of METH and fentanyl. The results indicated that the concurrent use of METH and fentanyl increased the acyl-ghrelin and total ghrelin levels in blood plasma. These findings suggest that the ghrelin system could be a potential pharmacological target for the treatment of substance use disorders caused by the combined use of METH and fentanyl (denoted as the METH-fentanyl polydrug below).

## 2. Results

The present research was devised to assess the effects of the METH-fentanyl polydrug on the circulating ghrelin in rats by determining blood concentrations of acyl-ghrelin, desacyl-ghrelin, and total ghrelin in comparison with these in an untreated/control group of rats.

### 2.1. Drug-Taking Behavior When Rats Intravenously Self-Administer the METH-Fentanyl Polydrug

**Figure 1:**
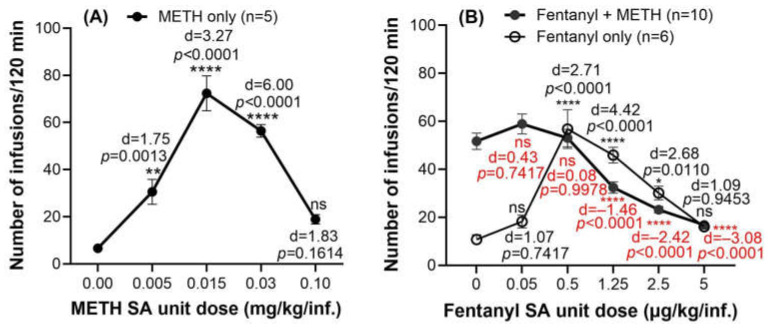
Dose–response curve for IVSA of METH alone (**A**) and fentanyl in combination with 0.03 mg/kg/inf. METH (**B**) in rats. Does-response curve for fentanyl only is also shown for comparison. The data are shown as the mean ± SEM. Statistical analysis: ns (not significant) *p* > 0.05, * p < 0.05, ** *p* < 0.01, and **** *p* < 0.0001; Effect size—Cohen’s d. Statistical data in red refer to the group of rats treated with the combined fentanyl and METH (the METH-fentanyl polydrug).

Numerous previous studies showed that Sprague-Dawley rats responded well to METH at a unit dose of 0.03 mg/kg/infusion in daily 2 h sessions under fixed ratio 1 (FR1) schedule of reinforcement, generating stable and consistent intravenous self-administration of METH in rats (IVSA) [19,26,27,28,29,30,31]. Our results for the dose–response curve from the intravenous self-administration (IVSA) studies of METH alone in rats tested under the same conditions (2 h IVSA under FR1 schedule) are consistent with this, as shown in Figure 1A. Therefore, to examine the drug-taking behavior of the polysubstance use by rats, the METH unit dose in our mixed drug stock was fixed as 0.03 mg/kg/infusion, with which the polydrug IVSA dose–response relationship was tested under the FR1 schedule of reinforcement within 120 min across a range of fentanyl doses of 0.0, 0.05, 0.5, 1.25, 2.5, and 5 µg/kg/infusion. As shown in Figure 1B and listed in Table 1, the number of polydrug infusions increased when decreasing the unit dose of fentanyl from 5 µg/kg/infusion to 0.5 µg/kg/infusion, but remained almost unchanged (51 to 53 infusions) when further decreasing the fentanyl unit dose from 0.5 µg/kg/infusion to zero (i.e., METH only, without fentanyl).

In the experiments discussed below, we chose to investigate the polysubstance use with a fixed composition percentage of the two drugs in a mixed drug solution, i.e., a combination of 0.0025 mg/kg/inf. fentanyl and 0.03 mg/kg/inf. METH to reflect effects from both fentanyl and METH. These unit doses were chosen for a couple of reasons. First, according to the weight percentage of fentanyl in the total weight of consumed drugs (i.e., a sum of consumed fentanyl and METH per kilogram of rat within 2 h), the fentanyl amount consumed by rats in the mixed drug solution varied from 0 to 14.3% (Table 1). Based on the fact that fentanyl ranged from 0.1% to 10% (≤10%) of the adulterated drug mixture in seized street illegal METH samples [32,33], the combination of 0.0025 mg/kg/inf. fentanyl and 0.03 mg/kg/inf. METH corresponds to a 7.7% fentanyl adulteration of METH. Additionally, according to a previously reported rat self-administration study [34] on the drug-vs-food choices of the unit doses for fentanyl and METH, the ED_50_ choice was 0.002 mg/kg/inf. (or 2.0 µg/kg/inf.) for fentanyl alone or 0.1124 mg/kg/inf. for METH alone. The unit dose of 0.03 mg/kg/inf. METH used in our study was very close to the ED_50_ choice (0.0336 mg/kg/inf.) and the unit dose of 0.0025 mg/kg/inf. fentanyl used in our study was also close to the ED_50_ choice of 0.002 mg/kg/inf. for fentanyl alone. However, further increasing the fentanyl dose to 0.005 mg/kg/inf. fentanyl (without changing the number of infusions) in this study, some rats showed severe toxicity signs, such as actively chewing front limbs with blood present on their limbs and mouths. So, the unit dose of 0.0025 mg/kg/inf. fentanyl is appropriate for the present study in terms of balancing the pharmacological effects and toxicity of fentanyl. 

### 2.2. Effects of the METH-Fentanyl Polydrug on Blood Plasma Concentrations of Ghrelin in Rats

**Figure 2:**
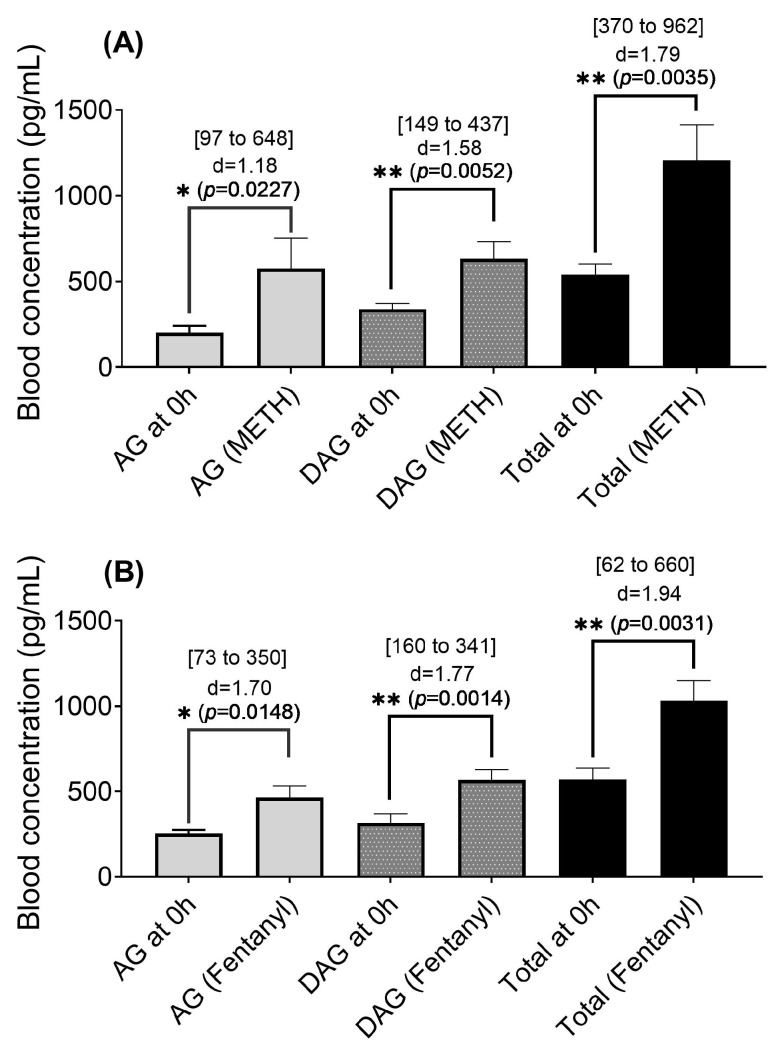
Blood concentrations of acyl-ghrelin (AG), desacyl-ghrelin (DAG), and total ghrelin (AG + DAG = Total) in rats (*n* = 6 per group) at time 0 h (pre-dose) and 2 h after the 2 h manual drug administration session in which each rat received 25 infusions of (**A**) METH at a unit dose of 0.03 mg/kg/infusion or (**B**) fentanyl at a unit dose of 0.0025 mg/kg/infusion. The data are shown as the mean ± SEM, along with the effect size (Cohen’s d) and 95% confidence interval (CI, in bracket) of the changes. Statistical significance (*t*-test): * *p* < 0.05 and ** *p* < 0.01.

**Figure 3:**
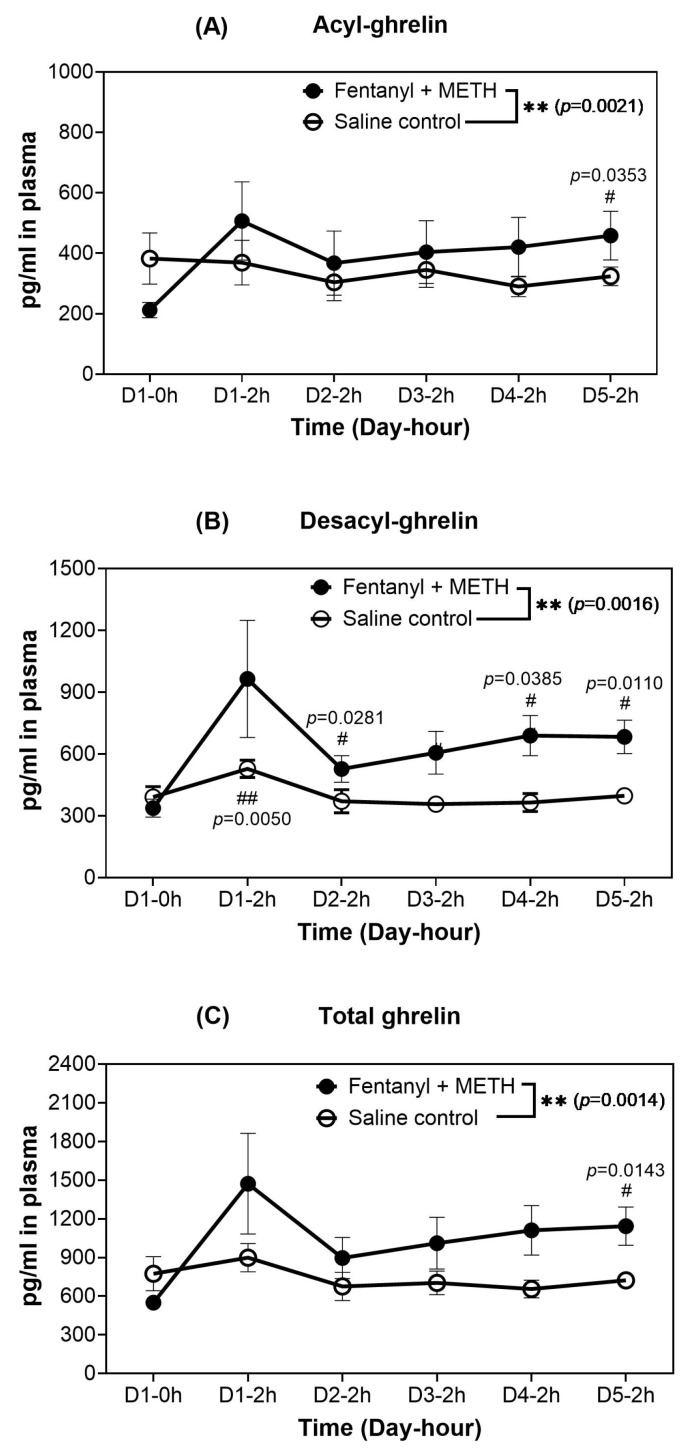
Blood concentrations of acyl-ghrelin, desacyl-ghrelin, and total ghrelin in rats (*n* = 6 per group) before the polydrug infusions on day 1 (pre-dose control), and immediately after 25 infusions of the METH-fentanyl polydrug (administrated evenly by humans in terms of the timing within 2 h through IV catheters, i.e., one infusion every ~4.8 min) on days 1 to 5: (**A**) acyl-ghrelin (AG); (**B**) desacyl-ghrelin (DAG); and (**C**) total ghrelin (AG + DAG = Total). The data are shown as the mean ± SEM. Statistical significance: one-way ANOVA using the Dunnett correction with post hoc analysis for comparison of the levels at various time points with the reference level on Day 1-0 h—# *p* < 0.05 and ## *p* < 0.01; paired *t*-test for the overall difference between the two groups of rats—** *p* < 0.01. See Table 2 for additional statistical data obtained from the *t*-tests for the changes from the baseline levels on Day 0–0 h to each time point after the polydrug infusion.

To assess the impact of the METH-fentanyl polydrug on the levels of acyl- and desacyl-ghrelin in blood, rats (*n* = 6/group) were administered manually with 25 infusions of the polydrug (i.e., 7.7% fentanyl-adulterated METH) within a two-hour session to rats through intravenous (IV) catheters inserted into the left femoral vein (the same as the ones used for the rat self-administration experiment mentioned below). The two-hour session started at about 9 am every morning during a period of five days (days 1 to 5, one session per day). Blood samples were collected right before the first session (pre-dose control on day 1 at time zero) and immediately after each session daily for analyzing plasma concentrations of acyl-ghrelin and desacyl-ghrelin. A similar procedure for the manual administration of 25 infusions was also carried out to examine the effects of individual drugs (METH or fentanyl alone), but for a single day only. The obtained ghrelin concentrations are shown in Figure 2 and Figure 3.

As shown in Figure 2, each individual drug (METH or fentanyl) significantly increased the blood levels of acyl-ghrelin, desacyl-ghrelin, and total ghrelin from time 0 h (pre-dose) to 2 h (the end of the 2 h drug administration session).

Concerning the impact of the METH-fentanyl polydrug, as shown in Figure 3, in comparison with the corresponding pre-dose levels (day 1 at time zero), the obtained plasma concentrations of acyl-ghrelin, desacyl-ghrelin, and total ghrelin increased on day 1 time 2 h after the rats received 25 infusions of the METH-fentanyl polydrug. Specifically, as seen in Figure 3A, the average concentration of acyl-ghrelin was 212 pg/mL on day 1 time 0 (the pre-dose level of the treatment group) and increased to 507 pg/mL on day 1 time 2 h (a 139% increase), then 368 pg/mL (a 74% increase), 404 pg/mL (a 91% increase), 420 pg/mL (a 98% increase), and 459 pg/mL (a 117% increase) at 2 h on days 2–5, respectively. The increase in acyl-ghrelin from the pre-dose level was statistically significant.

As shown in Figure 3B, the average concentration of desacyl-ghrelin was 338 pg/mL on day 1 time 0 (the pre-dose level of the treatment group) and increased to 965 pg/mL on day 1 time 2 h (a 186% increase), and then 528 pg/mL (a 56% increase), 607 pg/mL (a 80% increase), 690 pg/mL (a 104% increase), and 684 pg/mL (a 102% increase) at 2 h on days 2–5, respectively. The increase in desacyl-ghrelin from the pre-dose level was also statistically significant for all days at 2 h (Figure 3B). Understandably, when both the acyl- and desacyl-ghrelin concentrations increased, the total ghrelin concentrations also increased (Figure 3C). Overall, based on paired *t*-test as indicated in Figure 3, there were significant differences between the polydrug treatment group and the saline control group (*n* = 6) in acyl-ghrelin, desacyl-ghrelin, total ghrelin concentrations. So, the infusions of the METH-fentanyl polydrug significantly increased the acyl-ghrelin, desacyl-ghrelin, and total ghrelin concentrations in rats.

**Table 2:** Changes in blood concentrations of acyl-ghrelin, desacyl-ghrelin, and total ghrelin in polydrug-treated rats (*n* = 6 per group) at each time point (each day at 2 h) after the polydrug infusions from the corresponding baselines on Day 0-0 h.

Analyte	Day at 2 h	Mean of the Changes (pg/mL) from Day 0-0 h	Range of the Changes (pg/mL) *^a^*	95%CI of the Changes (pg/mL)	*p* (*t*-Test)	Effect Size (Cohen’s d)
AG	Day 1	295	8 to 563	68 to 521	0.0256	1.29
	Day 2	156	34 to 540	−11 to 323	0.0637	0.82
	Day 3	192	58 to 597	29 to 355	0.0343	1.04
	Day 4	209	53 to 575	55 to 362	0.0223	1.19
	Day 5	247	80 to 422	125 to 369	0.0053	1.68
DAG	Day 1	627	182 to 2066	40 to 1214	0.0453	1.26
	Day 2	190	81 to 401	101 to 278	0.0042	1.40
	Day 3	269	115 to 644	111 to 428	0.0104	1.38
	Day 4	352	156 to 733	174 to 530	0.0058	1.90
	Day 5	346	161 to 407	218 to 474	0.0016	2.17
Total ghrelin	Day 1	922	248 to 2740	140 to 1704	0.0345	1.35
	Day 2	346	118 to 941	98 to 593	0.0205	1.17
	Day 3	461	203 to 1241	148 to 775	0.0172	1.27
	Day 4	561	209 to 1308	232 to 889	0.0102	1.61
	Day 5	593	241 to 978	359 to 827	0.0021	2.15

*^a^* The smallest and largest changes in the concentrations within all the six polydrug-treated rats.

### 2.3. Blood Ghrelin Levels in Rats That Self-Administrated the METH-Fentanyl Polydrug

Rats (*n* = 10) implanted with IV catheters were trained to self-administer the METH-fentanyl polydrug under a schedule of fixed ratio 1 (FR1) and time out 5 s (TO5) in daily 2 h sessions with a total of 14 sessions. Additionally, to determine the pre-dose control concentrations of the acyl- and desacyl-ghrelin concentrations in rats, prior to the polydrug self-administration (SA) training, rats received 25 infusions of saline through uniformly manual administration within 2 h, followed immediately by blood collections.

**Figure 4:**
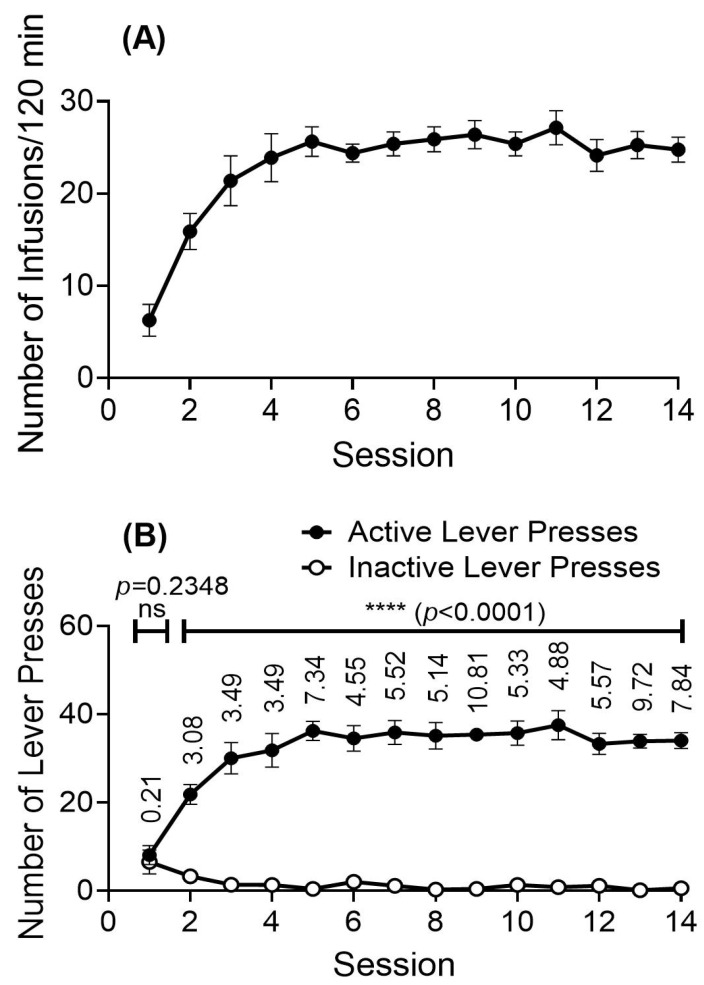
Responses of rats (*n* = 10) self-administering the METH-fentanyl polydrug solution (combined 0.03 mg/kg/inf. METH and 0.0025 mg/kg/inf. fentanyl) during the two-hour SA sessions under FR1TO5. (**A**) Number of the received infusions; (**B**) Number of presses on the active or inactive lever (Control). The data are shown as the mean ± SEM. Statistical analysis: *t*-test—ns, *p* = 0.2348 (Session 1) and ****, *p* < 0.0001 for each session during Sessions 2 to 14; Effect size—Indicated above the numbers of active lever presses are the effect size (Cohen’s d) values for the difference between the number of active lever presses and number of inactive lever presses.

According to the SA data shown in Figure 4, with the training METH-fentanyl polydrug solution (a combination of 0.03 mg/kg/inf. METH and 0.0025 mg/kg/inf. fentanyl), rats initially pressed more on inactive levers but quickly switched to active levers and acquired self-administration, as demonstrated by the increase in active lever responses and number of polydrug infusions during the 14 training sessions. Responses on the inactive lever were less than six on average for each session. After five sessions of SA training, the rats stably took an average of ~25 infusions daily for each training session. Blood samples were collected immediately after the 13th and 14th SA sessions for blood ghrelin level analyses, during which the 10 rats took an average of 26.2 and 25.4 polydrug infusions in the 13th and 14th SA sessions, respectively.

**Figure 5:**
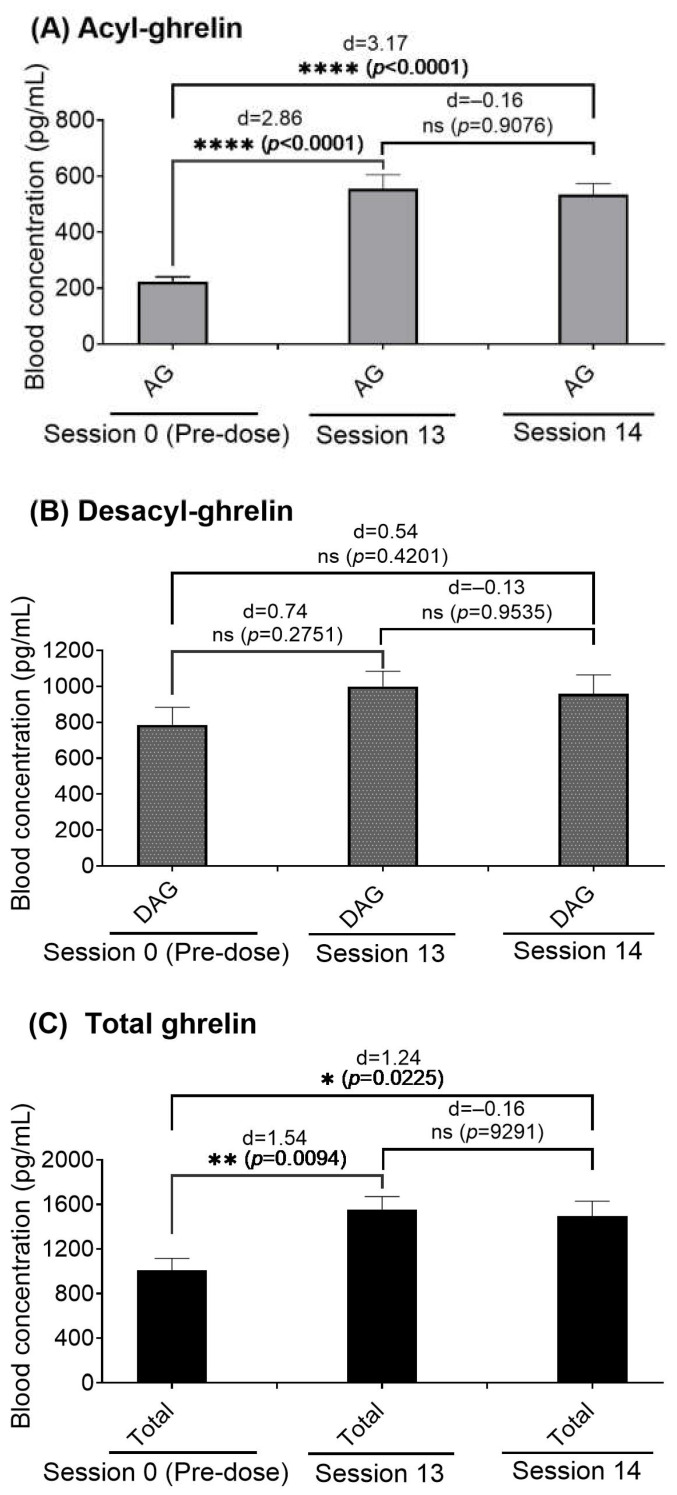
Measured blood concentrations of (**A**) acyl-ghrelin (AG), (**B**) desacyl-ghrelin (DAG), and (**C**) total ghrelin (AG + DAG = Total) immediately after Sessions 13 and 14 in comparison with the corresponding pre-dose baselines immediately after Session 0 (*n* = 10). The data are shown as the mean ± SEM, along with the effect sizes (Cohen’s d) of the differences between sessions. Statistical significance (one-way ANOVA using the Dunnett (recommended) correction with post hoc analysis): * *p* < 0.05, ** *p* < 0.01, and **** *p* < 0.0001.

As shown in Figure 5, the acyl-ghrelin, desacyl-ghrelin, and total ghrelin levels in rats (*n* = 10) increased by 130% (statistically significant), 24% (statistically insignificant), and 49% (statistically significant), respectively, after the 13th session, and increased 124% (statistically significant), 20% (statistically insignificant), and 44% (statistically significant), respectively, after 14th session. The acyl-ghrelin, desacyl-ghrelin, and total ghrelin concentrations after the 14th session were very close to the corresponding concentrations after the 13th session. The acyl-ghrelin and total ghrelin concentrations after the 13th or 14th session were significantly higher than the corresponding pre-dose concentrations on day 0.

## 3. Discussion

Ghrelin, as a hormone, is mainly produced in the stomach and circulates in the blood stream. Ghrelin crosses the blood–brain barrier (BBB) [35] via passive diffusion and/or transporter(s) [36] and mediates the rewarding and motivational properties of addictive substances through activating the ghrelin receptor, which is also known as the growth hormone (GH) secretagogue receptor (GHSR or GHS-R1a in the literature) [18,37,38,39,40,41]. Hence, the ghrelin system is recognized as a potential pharmacotherapeutic target for the treatment of drug abuse [42,43]. Extensive studies indicate that several types of individual drugs of abuse, including METH and cocaine, upregulate the ghrelin system and enhance cravings for drugs. However, the effects of fentanyl alone or the METH-fentanyl polydrug on the ghrelin system have not been investigated.

According to the data obtained in the present study, METH or fentanyl alone significantly increased the blood levels of acyl-ghrelin, desacyl-ghrelin, and total ghrelin. The outcome of the study with METH is consistent with the previously reported study on METH [23]; it is the first report demonstrating that the use of fentanyl also significantly increased the blood levels of acyl-ghrelin, desacyl-ghrelin, and total ghrelin.

Further, we examined the effects of the METH-fentanyl polydrug on circulating ghrelin levels in rats for the first time by performing two experiments. Previous studies on the ghrelin system in rats demonstrated that *n* = 6/group was sufficient to achieve the desired statistical significance [5,26]. So, we used rats with *n* ≥ 6 per group (*n* = 6 for experiment 1 and *n* = 10 for experiment 2) in this study. To obtain the accurate response of the living systems to the polydrug, we first manually administered specifically designed quantities (25 infusions within two hours per day) of the polydrug to rats during a period of five days (experiment 1). The number of infusions was consistent with the average number of infusions that rats earned during SA sessions (25 infusions in a 2 h session). In designing this study, we first established the METH dose–response curve in rats. These results indicated that 0.03 mg/kg/infusion was an appropriate METH dose to combine with fentanyl, based on the stability of responding and the number of infusions rats earned. We then presented rats with this unit dose of METH (0.03 mg/kg/infusion) paired with a varying unit dose of fentanyl, allowing the rats to self-determine the amount of drug they preferred (i.e., the number of infusions for each combination). As shown in Figure 1 and Table 1, rats took approximately 25 infusions on average when 0.03 mg/kg/infusion METH was mixed with 2.5 µg/kg/infusion fentanyl. Experiment 1 (the manual 25-infusion protocol) characterized the biochemical changes—reflected in circulating ghrelin levels—produced by passive administration of 25 infusions of the 7.7% fentanyl-adulterated METH. In contrast, experiment 2 (the self-administration protocol) demonstrated that, after establishing motivation and reward expectation for the fentanyl-adulterated METH, rats reliably self-administered a similar number of infusions and showed comparable changes in circulating ghrelin. According to the data obtained in experiment 1, the METH-fentanyl polydrug significantly increased blood levels of acyl-ghrelin, desacyl-ghrelin, and total ghrelin within the period of five days.

A further rat self-administration experiment (experiment 2) demonstrated that rats became addicted to the METH-fentanyl polydrug and stably took ~25 infusions of the polydrug daily. In this experiment, we were looking for the long-lasting effects of the METH-fentanyl polydrug on the ghrelin systems after the 13th and 14th SA sessions. Based on the data, the polydrug significantly increased the blood levels of acyl-ghrelin and total ghrelin in the rats after the 13th and 14th SA sessions compared to the pre-dose saline baselines after SA session 0. These results suggest that the elevation of circulating acyl-ghrelin and total ghrelin levels correlates to the polydrug addiction behavior. Notably, the polydrug did not statistically significantly increase the desacyl-ghrelin level after the 13th and 14th SA sessions, suggesting that the observed polydrug-induced increase in desacyl-ghrelin level in experiment 1 might last for only a few days. Further studies should be performed to fully understand this issue in the future.

The two experiments (experiments 1 and 2) have consistently demonstrated that the METH-fentanyl polydrug can significantly increase the blood levels of acyl-ghrelin and total ghrelin in rats. The observed polydrug-induced increases in acyl-ghrelin and total ghrelin in rat blood are expected to occur in the corresponding rat brain according to previous reports [26,44]. However, we were unable to measure the brain ghrelin levels in living animals, which is a limitation for this study. Without directly measuring the brain ghrelin concentrations, we cannot affirm the changes in brain ghrelin levels. So, it would be interesting to develop a new technology with the capability to simultaneously measure the blood and brain ghrelin levels in living animals in the future.

Moreover, the results from the current study in rat models also provide valuable insights for future human studies. Notably, rats and humans share fundamental brain reward pathways, making rats a valuable model for investigating the neurobiological mechanisms underlying addiction to stimulants and opioids. Rats also exhibit complex behaviors that parallel human addiction, including compulsive drug-seeking and the co-use of stimulants and opioids. By examining how these drugs interact with the system in a well-designed rat model, researchers can gain critical insights into human addiction and advance the development of more effective interventions and treatment strategies for the ongoing public health crisis. In this study, we demonstrated that the METH-fentanyl polydrug elevates blood ghrelin levels—an effect that has a high possibility of occurring in humans. Additionally, our findings indicate that the fentanyl proportion in the METH–fentanyl polydrug should remain low—ideally below 10%—in any future human clinical trials to ensure participant safety. More broadly, fentanyl dose selection for clinical studies must consider multiple factors, including interspecies differences in pharmacokinetics and tolerability. For instance, the elimination half-life of fentanyl is approximately 50 min in rats [45] but it ranges from 219 to 853 min (~3.6 to ~14.2 h) in humans [46]. The ultimately selected fentanyl dose for such studies should be sufficient to achieve the intended clinical effects while remaining within a safety margin appropriate for human participants.

In summary, this study has demonstrated for the first time that the METH-fentanyl polydrug, or fentanyl use alone, significantly increased plasma acyl-ghrelin (the active form of ghrelin) and total ghrelin concentrations. This finding suggests that the ghrelin system may serve as a potentially promising therapeutic target for the development of a treatment for the METH-fentanyl polydrug as well as fentanyl use alone, although further target validation studies using possible chemical and/or biological probes to downregulate ghrelin will be required to test and validate the potentially promising target. Downregulation of ghrelin signaling may be achieved by administrating a potent and selective antagonist of receptor GHS-R1a or a ghrelin deacylase [47,48], which can convert acyl-ghrelin to desacyl-ghrelin. For the former, administration of a potent and selective antagonist of receptor GHS-R1a may help to competitively block GHS-R1a binding with ghrelin and block the ghrelin-induced activation of GHS-R1a and, thus, attenuate the reward effect of the polydrug. For the latter, it is well known that only acyl-ghrelin can bind and activate receptor GHS-R1a, and desacyl-ghrelin is inactive for GHS-R1a. Administration of an exogenous ghrelin deacylase may help to downregulate the acyl-ghrelin level itself and, thus, indirectly decrease the acyl-ghrelin-induced activation of GHS-R1a—an alternative strategy to attenuate the reward effect of the polydrug or fentanyl use alone. It would be interesting to explore these potentially interesting therapeutic strategies in future studies.

## 4. Materials and Methods

### 4.1. Animals and Drugs

Male Sprague-Dawley rats (250–270 g) were ordered from Harlan (Indianapolis, IN, USA), allowed ad libitum access to food and water all the time except for the time of the experimental test, and allowed acclimatize for one week before the experiment. All animals (received as two rats per cage and single-caged after the surgery) were maintained on a 12 h light/12 h dark cycle. All experiments were performed during the light phase of the light/dark cycle in accordance with the animal protocol approved by the IACUC (Institutional Animal Care and Use Committee) at the University of Kentucky. All self-administration sessions started around 9 a.m. (+)-Methamphetamine hydrochloride (METH) and fentanyl hydrochloride were purchased from Sigma (Cream Ridge, NJ, USA) and dissolved in saline. Acyl-ghrelin enzyme-linked immunosorbent assay (ELISA) kit (#10006307) and desacyl-ghrelin ELISA kit (#10008953) were purchased from Cayman Chemical Company (Ann Arbor, MI, USA). A potassium phosphate buffer (0.1 M pH 7.4 with 10 mM p-hydroxymercuribenzoic acid and 1.2% NaOH (*v*/*v*)) and an EDTA solution (15 mg/mL, pH 8.0) were prepared for ghrelin sampling.

All the in vivo experiments were performed following the Guide for the Care and Use of Laboratory Animals as adopted and promulgated by the National Institutes of Health (NIH), and were consistent with the ARRIVE (Animal Research: Reporting of In Vivo Experiments) guidelines (https://arriveguidelines.org; last accessed 5 December 2025). The animal protocol was approved by the University of Kentucky’s Institutional Animal Care and Use Committee (IACUC).

### 4.2. Apparatus

Intravenous (IV) self-administration (IVSA) and manually administered, repeated intravenous injections were performed using the same set of operant conditioning chambers (29.5 cm L × 24.8 cm W × 18.7 cm H) ordered from Med Associates (St Albans, VT, USA) in the same procedure room, as described in our previous report in another study [49]. Briefly, each SA chamber was equipped with two retractable steel response levers that were connected to a computer system controlled by the MED-PC 4.2 software to program/control the experiments and record the SA data. Within the Med Associates system, each chamber was coupled with a food pellet (#F0021 from Bio-Serv, Flemington, NJ, USA) dispenser and connected to a variable rate syringe pump (#PHM-100VS) delivering the substance/saline solution via polyurethane tubing (0.025 × 0.055 in) ordered from In-stech Laboratories Inc. (Plymouth Meeting, PA, USA). The polyurethane tubing was connected to a liquid swivel and spring tether set (#VAH95T ordered from In-stech Laboratories Inc. as well).

### 4.3. Intranvenous Catheterization Surgery

Prior to surgery, the rats were pre-trained for lever pressing with dustless diet pellet (#F0021 from the Bio-Serv) to acquire 40 food pellets within 2 h. At the surgery time, the rats weighed 300–400 g. IV catheterization surgery was carried out aseptically following a procedure described in our previous report [49]. Briefly, the IV catheter (#C30PU-RFV1418 from In-stech Laboratories, Inc.) was inserted into a femoral vein under anesthesia using isoflurane. The internal end of catheter was secured in a position anchored with nonabsorbable surgical sutures (# 51-7623 Size 3-0 from Harvard Apparatus, Holliston, MA, USA), whereas the other (distal) end of the catheter on the back was connected to a support harness (#VAH95AB from In-stech Laboratories, Inc.). The harness was put around the scapula. A small plastic cap was used to seal the base of the catheter to remain sterile and protect the catheter. After surgery, rats were administered with 5 mg/kg carprofen (SC) and 20 mg/kg cefazoline (IV) once per day for three and seven days, respectively. The catheters were flushed daily by using heparinized saline. Rats were given 7–14 days of recovery before use for experiment.

### 4.4. Self-Administration Training and Dose–Response Curve

Rats with IV catheters were trained to self-administer a drug solution under a schedule of fixed ratio-1 (FR1) and time out 5 s (TO-5) in daily 2 h sessions. Under the FR1 schedule, a rat needed to press the active lever (the right lever) once to receive a unit dose of training drug solution. Responding on the left lever was recorded, but had no programmed consequence (inactive). Inactive lever presses, compared with active lever presses, were used as a control to determine the drug’s effect on active behavior (drug intake). A 5.0 mL syringe (SKU:301027; BD Medical Systems Luer-Lok Syringe) containing a drug solution (METH alone or fentanyl alone or the METH-fentanyl polydrug), was positioned tightly on the pump, which was set at RPM 5 with a flow rate of 0.7356 mL/min. For example, for a 350 g rat to obtain an infusion of 0.03 mg/kg/inf. METH with 0.0025 mg/mL/inf. fentanyl, the pump operates for 5.7 s. A stable baseline was achieved after the rats acquired a stable response in the 120 min session, i.e., with a variation of less than three infusions over the last three sessions. Various METH unit doses (0.00, 0.005, 0.015, 0.03, and 0.10 mg/kg/inf.) were used to obtain the dose–response curve of METH IVSA in rats (*n* = 5); various fentanyl unit doses (0, 0.05, 0.5, 1.25, 2.5, and 5 µg/kg/inf.) were used to obtain the dose–response curve of fentanyl IVSA in rats (*n* = 6); the dose–response curve of fentanyl IVSA with a fixed dose of 0.03 mg/kg/inf. METH (*n* = 10) was obtained by conducting 2 h IVSA testing sessions using various fentanyl unit doses (0, 0.05, 0.5, 1.25, 2.5, or 5 µg/kg/inf.) with a fixed unit dose of METH (0.03 mg/kg/inf.). For each drug unit dose, one to three training sessions were carried out, and all obtained data were used to generate a dose response curve.

### 4.5. Effects of Manually Administered METH-Fentanyl Polydrug on Ghrelin Levels

Two groups of rats (*n* = 6/group) were used to examine the short-term effects of the individual drug alone (METH or fentanyl) on the ghrelin system of rats. Unearned METH or fentanyl solution was injected repeatedly into rats through intravenous catheters by using the “operation” function of the pump to deliver the drug solution during the two-hour SA session (25 infusions in two hours in total, with one infusion every ~4.8 min). The unit dose of METH used in the METH experiment was 0.03 mg/kg/infusion. The unit dose of fentanyl used in the fentanyl experiment was 2.5 µg/mL/infusion. Plasma samples for ghrelin analyses were collected before (at 0 h) and immediately after the 2 h session (at 2 h). 74 µL blood samples collected for analyzing acyl-ghrelin and deascyl-ghrelin were mixed with 20 µL EDTA-containing potassium phosphate buffer solution (prepared with 10 µL potassium phosphate buffer and 10 µL EDTA solution). The mixture was centrifuged at 3500 rpm at 4 °C for 15 min, and the supernatant was aliquoted into two separate tubes: one for acyl-ghrelin ELISA analysis, and the other for desacyl-ghrelin ELISA analysis. All samples were stored at −20 °C prior to ELISA. Samples were kept on ice between collection and centrifuge.

Two other groups of rats (randomly assigned with *n* = 6 for the saline group and *n* = 6 for the drug group) were tested for the short and long-term effects of the polydrug (i.e., the METH-fentanyl polydrug) on acyl-ghrelin and desacyl-ghrelin levels in rats. Unearned polydrug solution was injected repeatedly into rats through intravenous catheters by using the “operation” function of the pump to deliver the polydrug solution during the two-hour SA session (25 infusions in two hours). The number of infusions was consistent with the average number of infusions that the rats earned during the METH-fentanyl polydrug SA sessions (25 infusions in 2 h session). The dose used in this experiment was METH—0.03 mg/kg/inf. and fentanyl—2.5 ug/mL/inf. Plasma samples for ghrelin analysis were collected immediately after each 2 h session. Additionally, pre-dose blood samples were collected on day 1 immediately before the polydrug infusions (at 0 h) as well. The procedure for sample preparation was the same as described above. All samples were stored at −20 °C prior to ELISA.

### 4.6. Ghrelin Concentrations in Rats That Intravenously Self-Administered the METH-Fentanyl Polydrug

Upon full recovery from the surgery, a group of rats (*n* = 10) was used to examine the effects of the polydrug IVSA on acyl-ghrelin and desacyl-ghrelin concentrations in rats, especially comparing ghrelin levels on pre-drug use and post addiction of the METH-fentanyl polydrug. Rats were manually administered 25 saline infusions prior to the first IVSA session (one infusion every ~4.8 min within 2 h). Then, the rats were trained to self-administer the METH-fentanyl polydrug solution under a schedule of FR-1 and TO-5 with a unit dose of METH—0.03 mg/kg/inf. and fentanyl—0.0025 mg/mL/inf. for 14 sessions. Each session started at 9 am and lasted 2 h every day. Ghrelin samples were collected at the saline session and at the end of the 13th and 14th sessions using the exact same procedures as in the previous experiments. Concerning the timing of the sessions, the pre-dose saline session (Session 0) was performed three days (Day -3) before the first polydrug session on Day 0 (Session 1), followed by Session 2 (Day 5), Session 3 (Day 7), and Session 4 (Day 8), and then daily sessions from Sessions 5 to 14 with only one day of (Day 17) break between Sessions 10 and 11 (i.e., daily sessions from Session 5 on Day 11 to Session 10 on Day 16 and Session 11 on Day 18 to Session 14 on Day 21).

### 4.7. Sample Analysis

Acyl- and desacyl-ghrelin concentrations in the blood samples were analyzed using the corresponding ELISA kits (96 well plates) according to the manufacturer’s instructions. To avoid matrix effects, 12 µL of ghrelin supernatant samples were diluted at 1:10 with the EIA buffer. 100 µL of the dilutes were dispensed to wells and mixed with 100 µL ghrelin tracer solution. After 3 h of incubation, the plates were washed, and optimal development was obtained by adding 200 µL of Ellman’s reagent to each well. The absorbance was measured by reading the plate at a wavelength of 405 nm (yellow color) and compared to the absorption for the standard samples within the same plate to calculate the concentration of ghrelin in diluted unknown samples.

### 4.8. Statistical Analysis

A GraphPad Prism 10 provided by GraphPad Software (La Jolla, CA, USA) was used to analyze the data in this study. One-way analysis of variance (ANOVA) using the Dunnett (recommended) correction method with post hoc analysis was used to analyze the concentration data at various time points in comparison with the corresponding pre-dose baselines. The difference between two groups or two different time points within the same group was analyzed by using the paired *t*-test, with each time point or each rat for a pair. A difference was considered statistically significant when *p* < 0.05 (* or # *p* < 0.05; ** or ## *p* < 0.01; *** or ### *p* < 0.001; and **** or #### *p* < 0.0001). 

## Figures and Tables

**Table 1:** Number of infusions during the dose-repose testing for the METH-fentanyl polydrug in rats, and the corresponding consumed fentanyl and METH.

Fentanyl (µg/kg/Infusion)	0.0	0.05	0.5	1.25	2.5	5.0
Number of infusions	52	59	53	32	23	17
Consumed METH (mg/kg)	1.551	1.767	1.590	0.972	0.695	0.506
Consumed fentanyl (mg/kg)	0.000	0.003	0.027	0.041	0.058	0.084
Ratio of fentanyl to METH (%)	0.000	0.167	1.667	4.167	8.333	16.667
Ratio of fentanyl to total amount of drugs (%)	0.000	0.166	1.639	4.000	7.692	14.286

## Data Availability

The original contributions presented in this study are included in the article. Further inquiries can be directed to the corresponding author(s).
